# Hepatic Steatosis and Thyroid Function Tests in Overweight and Obese Children

**DOI:** 10.1155/2013/381014

**Published:** 2013-02-03

**Authors:** L. Pacifico, E. Bonci, F. Ferraro, G. Andreoli, S. Bascetta, C. Chiesa

**Affiliations:** ^1^Department of Pediatrics, Sapienza University of Rome, Viale Regina Elena, 324 00161 Rome, Italy; ^2^Institute of Translational Pharmacology, National Research Council, 00133 Rome, Italy

## Abstract

*Objectives*. Associations between thyroid function and nonalcoholic fatty liver disease (NAFLD) are unknown in childhood. Thus, the aim of the present study was to investigate in 402 consecutive overweight/obese children the association between thyroid function tests and hepatic steatosis as well as metabolic variables. *Methods*. Hepatic steatosis was diagnosed by ultrasound after exclusion of infectious and metabolic disorders. Fasting serum samples were taken for determination of thyroid function (TSH, FT4, and FT3), along with alanine aminotransferase (ALT), lipid profile, glucose, insulin, and insulin resistance (IR). *Results*. Eighty-eight children (21.9%) had TSH above the normal range (>4.0 mIU/L). FT3 and FT4 were within the reference intervals in all subjects. Elevated TSH was associated with increased odds of having hepatic steatosis (OR 2.10 (95% CI, 1.22–3.60)), hepatic steatosis with elevated ALT (2.42 (95% CI, 1.29–4.51)), hypertriglyceridemia, elevated total cholesterol, and IR as well as metabolic syndrome (considered as a single clinical entity), after adjustment for age, gender, pubertal status, and body mass index-SD score (or waist circumference). *Conclusions*. In overweight/obese children, elevated TSH concentration is a significant predictor of hepatic steatosis and lipid and glucose dysmetabolism, independently of the degree of total and visceral obesity.

## 1. Introduction

Over the last two decades, the rise in the prevalence rates of overweight and obesity may explain the emergence of nonalcoholic fatty liver disease (NAFLD) as the leading cause of liver disease worldwide [1]. NAFLD, a novel component of metabolic syndrome (MetS) [2], is a spectrum of fat-associated liver conditions that can result in end-stage liver disease and the need for liver transplantation [1, 3]. Simple steatosis, or fatty liver, occurs early in NAFLD and may progress to nonalcoholic steatohepatitis (NASH), fibrosis, and cirrhosis with increased risk of hepatocellular carcinoma [1, 3]. 

Several previous studies have addressed the association between thyroid function and NAFLD [4–10]. The results of these studies support an association between overt or subclinical thyroid disease and NAFLD. This seems to be biologically plausible given the common clinical and biochemical features of central obesity, insulin resistance (IR), hypertension, hypertriglyceridemia, and lipid peroxidation observed in both NAFLD and hypothyroidism [5, 11]. However, confirmation and further characterization of clinical data supporting this association are needed. To our knowledge, none of the previous studies have investigated the relationship between hepatic steatosis and thyroid function in childhood. Because small abnormalities in thyroid function are common, thyroid function may importantly influence the prevalence of NAFLD in a population of overweight/obese children and adolescents. We therefore tested the hypothesis that even slightly elevated serum levels of TSH indicating mild thyroid failure might be associated with an increased risk for NAFLD. To this end we explored in overweight/obese children with no clinical, autoantibody, and ultrasonographic evidence of thyroid disease the association between thyroid function tests (thyroid-stimulating hormone (TSH), and free thyroxine (FT4), free triiodothyronine (FT3)) and hepatic steatosis as well as MetS and its parameters. 

## 2. Materials and Methods

### 2.1. Study Subjects

This is a cross-sectional study carried out at the Hepatology outpatient Clinic of the Department of Pediatrics, Sapienza University of Rome, Italy, between April 2010 and September 2012. The children included were all those being overweight (body mass index (BMI) higher than the age- and sex-specific 85th percentile) or obese (BMI higher than the age- and sex-specific 95th percentile) [12] aged 6–16 years. However, the following subjects were excluded: (a) those with renal disease; type 1 or 2 diabetes; any condition known to influence body composition, insulin action, or insulin secretion (e.g., glucocorticoid therapy, overt hypothyroidism, and Cushing's disease); (b) those with known thyroid disease, current or past history of thyroid hormone or antithyroid drug intake, thyroid alterations in volume and morphology at ultrasound, and positive antithyroglobulin (anti-TG) and anti-thyroperoxidase (anti-TPO) antibodies; (c) those with any laboratory or clinical evidence suggesting an alternate or coexistent underlying chronic liver disease including hepatic virus infections (Hepatitis A–E and G, cytomegalovirus, and Epstein-Barr virus), autoimmune hepatitis, metabolic hepatic disease, *α*-1-antitrypsin deficiency, cystic fibrosis, Wilson's disease, hemochromatosis, and celiac disease; and (d) those with history of alcohol consumption and smoking (where appropriate).

All study participants underwent physical examination including measurements of weight, standing height, BMI, waist circumference (WC), determination of the stage of puberty, degree of obesity, and systolic blood pressure (BP) and diastolic BP. The pubertal stage was categorized into two groups (prepubertal: boys with pubic hair and gonadal stage I, and girls with pubic hair and breast stage I; pubertal: boys with pubic hair and gonadal stage ≥II and girls with pubic hair stage and breast stage ≥II). WC was obtained at the midpoint between the lowest rib and the iliac crest. The measurement was made at the end of a normal expiration while the subjects were in a standing position. The degree of obesity was quantified using Cole's least mean-square method, which normalizes the skewed distribution of BMI and expresses BMI as a SD score (SDS). This measure gives age- and gender-specific estimates of the distribution median, the coefficient of variation, and the degree of skew by a maximum likelihood fitting technique [12]. Systolic and diastolic BP were measured twice at the right arm after a 10 min rest in the supine position by using an automated oscillatory system (Dinamap Vital Signs Monitor, Model 1846 SX; Criticon Incorporated, Tampa, FL, USA).

 The study was approved by the Hospital Ethics Committee, and informed consent was obtained from subjects' parents prior to assessment.

### 2.2. Laboratory Investigations

Blood samples were taken from each subject, after an overnight fast, for estimation of glucose, insulin, total cholesterol and high-density lipoprotein cholesterol (HDL-C), triglycerides, alanine aminotransferase (ALT), gamma-glutamyl transferase (GGT), TSH, FT4, FT3, and anti-TG and anti-TPO antibodies. All analyses were conducted by COBAS 6000. TSH, FT3, FT4, anti-TG and anti-TPO antibodies, and insulin were measured on cobas e 601 module (Electrochemiluminescence Technology, Roche Diagnostics), while the remaining analytes on cobas c 501 clinical chemistry module (Photometric Technology), according to the instructions of the manufacturer. 

TSH was considered normal if it was between 0.4 and 4.0 mIU/L [13–16]. The reference intervals for FT3 and FT4 were 2.70–5.20 pg/mL and 0.80–1.90 ng/dL, respectively. Threshold values for anti-TG and anti-TPO antibodies were 115 and 34 IU/mL, respectively.

### 2.3. Definitions

Ultrasound examinations of the liver and of the thyroid were performed in real time using an Aplio XV (Toshiba Medical Systems) with 3.5–5-MHz convex transducers and tissue harmonics. Hepatic steatosis was defined by an appearance of hyperechoic liver parenchyma with tightly packed fine echoes and posterior beam attenuation. At ultrasound, thyroid size and morphology were evaluated with a high-resolution 7.5 MHz linear transducer, with the subjects sitting and their necks slightly extended. Thyroid volume was defined as enlarged according to the reference values for age, gender, and body surface area [17]. Thyroid tissue echogenicity was evaluated in a longitudinal scan of the thyroid lobes by a standardized comparison with the echogenicity of the adjacent muscles: sternohyoideus, sternothyroideus, and sternocleidomastoideus. Ultrasound scans were performed by a well-trained ultrasonographist who was unaware of the clinical and laboratory data.

For the American Heart Association (AHA) [18], MetS is diagnosed in the presence of any three of the following five constituent risks: central obesity as determined by WC, hypertension, low HDL-C values, elevated triglyceride values, and glucose impairment. We used the pediatric AHA definition [19], which is based on the AHA adult definition but uses pediatric reference standards for BP, WC, triglycerides, and HDL-C. Thus, in our study, central obesity was defined as a WC ≥90th percentile for age and gender; hypertriglyceridemia as triglycerides ≥90th percentile for age and gender; low HDL-C as concentrations ≤10th percentile for age and gender; elevated BP as systolic or diastolic BP ≥90th percentile for age, gender, and height percentile; and impaired fasting glucose as glucose ≥5.6 mmol/L. IR was determined by a homeostasis model assessment of insulin resistance (HOMA-IR) [20]. We considered HOMA-IR values ≥90th percentile for age and gender of those previously observed in a local population of healthy normal-weight children as an indicator of IR [21]. 

### 2.4. Statistical Analysis

Statistical analyses were performed using the SPSS package. Data are expressed either as frequencies or means with 95% confidence intervals (CIs). Distributions of continuous variables were examined for skewness and kurtosis and were logarithmically transformed, when appropriate. Geometric means are reported for TSH, FT3, FT4, total cholesterol and HDL-C, triglycerides, insulin, and HOMA-IR values. Differences between groups were tested for significance using independent sample *t*-test for quantitative variables, and chi-square test for qualitative variables. Pearson's correlation and linear regression coefficients were used to examine the relationship between variables. Logistic regression analysis was performed to determine the independence of the association of TSH levels with hepatic steatosis, hepatic steatosis with elevated ALT, and metabolic variables, after adjustments for age, gender, pubertal status, and BMI-SDS (or WC) as well as FT3 and FT4 concentrations. Relationships between TSH levels and hepatic steatosis were also examined after adjustment for anthropometric and metabolic variables including hypertriglyceridemia, hypercholesterolemia, and insulin resistance. In the fully adjusted regression model, FT3 and FT4 levels were also included. A *P* value of less than 0.05 was considered statistically significant.

## 3. Results

### 3.1. Clinical and Laboratory Features of Study Population

The study group consisted of a consecutive series of 402 children fulfilling the selection criteria. Ninety-seven and 305 were overweight and obese, respectively. In Table 1 clinical, metabolic, and hormonal characteristics of overweight/obese children according to TSH level are summarized. Eighty-eight subjects (21.9%) had TSH above the normal range (>4.0 mIU/L). FT3 and FT4 were within the reference intervals in all subjects. 

Compared to individuals with normal TSH, subjects with hyperthyrotropinemia showed significantly higher ALT, GGT, total cholesterol, triglycerides, insulin, and HOMA-IR values. Children with TSH ≤ 4.0 mIU/L and those with TSH over the normal range did not significantly differ in values of free thyroid hormones as well as in age, gender, pubertal status, or degree of total and central adiposity.

### 3.2. Thyroid Function Tests in relation to NAFLD and Metabolic Variables

Serum TSH concentrations were significantly higher in the 144 children with fatty liver compared to the 258 children with no fatty liver (3.20 (95% CI, 2.95–3.36) versus 2.65 (95% CI, 2.51–2.80); *P* < 0.01). The children with hepatic steatosis did not differ significantly from those with no hepatic steatosis in respect of FT3 (4.35 (95% CI, 4.26–4.48) versus 4.30 (95% CI, 4.14–4.40)) and FT4 (1.17 (95% CI, 1.13–1.21) versus 1.21 (95% CI, 1.17–1.24)). 

As shown in Table 2, elevated TSH concentrations were significantly associated with an increased prevalence of hepatic steatosis, and hepatic steatosis with elevated ALT concentrations (serum ALT >25 IU/L for boys and >22 IU/L for girls [22]). Among the metabolic variables, the prevalence of hypertriglyceridemia, IR, elevated total cholesterol, and MetS (considered as a single clinical entity) was significantly higher in the 88 subjects with elevated TSH concentrations than in the 314 subjects with normal TSH concentrations.  Table 3 shows the multivariate-adjusted associations between TSH and hepatic steatosis, as well as metabolic variables. High TSH was associated with an increased odds of having hepatic steatosis, hepatic steatosis with elevated ALT, hypertriglyceridemia, elevated total cholesterol, and IR as well as MetS, after adjustment for age, gender, pubertal status, and BMI-SDS (or WC) as well as FT3 and FT4. When a stepwise multivariate regression analysis included variables such as age, gender, pubertal status, BMI-SDS, hypertriglyceridemia, hypercholesterolemia, IR, and FT3 and FT4, the association between hepatic steatosis and TSH levels remained statistically significant (OR, 1.96 (95% CI, 1.10–3.75); *P* < 0.05). In this model, other covariates independently associated with hepatic steatosis were hypertriglyceridemia (2.73 (95% CI, 1.51–4.92); *P* < 0.01) and IR (2.37 (95% CI,  1.38–4.06); *P* < 0.01).

No association was found between FT4 and hepatic steatosis as well as MetS and its parameters after controlling for age, gender, pubertal status, and BMI-SDS (or WC).

## 4. Discussion

To the best of our knowledge, our study is the first to assess the relationships between thyroid function and hepatic steatosis in a large consecutive series of young participants. First, we found that in overweight/obese children and adolescents hyperthyrotropinemia was significantly associated with hepatic steatosis, after adjustments for age, gender, pubertal status, and degree of obesity. Second, we also found a significant association between elevated TSH levels and metabolic variables (including hypertriglyceridemia and IR which are usually altered in fatty liver). Finally, multivariate regression analysis showed that mild abnormalities in thyroid function were associated with hepatic steatosis independently of metabolic risk factors. 

Previous studies have investigated the association between NAFLD (or surrogate markers of NAFLD) and thyroid function but solely in adult or elderly patients [4–10, 23]. The study by Targher et al. involving a large cohort of unselected adult outpatients was the first to report a strong association between thyroid function tests and serum liver enzyme activity concentrations [23]. In particular, Targher et al. found a significant positive relationship between serum TSH, ALT, and GGT activities throughout the normal and high TSH ranges, and a similar inverse relationship between FT4 and serum liver enzyme activity concentrations [23]. Notably, these results did not change after adjustment for a broad spectrum of potential confounders, such as gender, age, fasting glucose and lipid parameters. Unfortunately, that study did not provide information on medication use, lifestyle characteristics (i.e., daily alcohol consumption), obesity status, and degree of insulin resistance among study participants. The recent data from a large-scale population-based study [9], the Study of Health in Germany (SHIP), partly agree with those from Targher et al. [23]. In the SHIP study, there was a significant inverse association between FT4 concentration and hepatic steatosis (defined by the presence of a hyperechogenic ultrasound pattern of the liver and increased ALT concentrations), whereas TSH and FT3 concentrations were not consistently associated with hepatic steatosis, thus suggesting that overt but not subclinical hypothyroidism (SH) might be associated with hepatic steatosis [9]. In a cross-sectional study performed among 878 euthyroid elderly Chinese who took their annual healthy examination, the prevalence rate of ultrasound-diagnosed NAFLD showed an increasing trend as serum TSH level increased, while the rate showed a decreasing trend as serum FT4 level increased [6]. Finally, in a recent cross-sectional study involving 4,648 health check-up subjects [7], Chung et al. found that the prevalence of ultrasound-diagnosed NAFLD and abnormal ALT levels increased steadily with increasing grades of hypothyroidism (for NAFLD, subclinical: 29.9% and overt: 36.3%; for abnormal ALT, 20.1% and 25.9%, *P* < 0.001, resp.). SH was defined by Chung et al. as a serum TSH level over 4.1 mIU/L with normal FT4 concentration (0.7–1.8 ng/dL), while overt hypothyroidism as a FT4 level less than 0.7 ng/dL. An 1 IU/L increase of natural logged TSH was associated with a 20% increase in the prevalence of NAFLD, independently of known risk factors such as age, gender, BMI, WC, triglyceride, HDL-C, hypertension, and diabetes [7]. 

The published literature investigating the association between thyroid function and NAFLD has also included patients with biopsy-proven NAFLD. Liangpunsakul and Chalasani retrospectively found that the prevalence of hypothyroidism was significantly higher in adult patients with NASH when compared with age-, gender-, race-, and body weight-matched adults without liver disease attending the general medical clinics [4]. In that study, however, data on thyroid function tests were not available and cases were defined solely based on use of synthetic T4 replacement therapy, therefore suggesting a high frequency of participants with overt hypothyroidism. Likewise, in a case control study involving 256 adult patients with NAFLD and 430 age-, gender-, race-, and BMI-matched adult outpatients with normal liver tests and no evidence of acute or chronic liver disease, Pagadala et al. retrospectively found that subjects with hypothyroidism were 2.1 (95% CI, 1.1–3.9, *P* = 0.02) and 3.8 (95% CI, 2–6.9, *P* < 0.001) times more likely to have NAFLD and NASH, respectively [8]. After adjusting for diabetes, hypertension, BMI, and hyperlipidemia, subjects with hypothyroidism were still found to be 2.1 times (95% CI, 1.1–3.9, *P* = 0.022) more likely to have NAFLD than those without hypothyroidism. In addition, hypothyroidism was significantly higher in patients with NASH compared to those with no NASH [8]. However, that study again did not include laboratory data regarding thyroid function, and subjects were defined as having hypothyroidism if they carried a clinical diagnosis of hypothyroidism and were on thyroid replacement therapy. Yet, in a study involving 69 euthyroid adult patients with primary NAFLD [10], Carulli et al. retrospectively found that when biopsy-proven NASH patients were compared to those with no NASH, high TSH levels, independently of MetS and IR, were predictors of NASH, but the possibility of selection bias was raised in that study as well as in the previous ones [4, 5, 8] because of liver biopsy.

Against that background, the strengths of our study are the careful clinical characterization of the participants with their risk factor profiles, and by pediatric standards a fairly large study sample. Compared to previous studies [4–10, 23], our data differ in several design features including demographic and clinical characteristics (young versus adult or older participants with more advanced forms of the disease), inclusion and exclusion criteria, data collection (prospective versus retrospective), study selection (consecutive versus nonconsecutive series), population details (sufficient versus insufficient), study design (cohort versus case control), or participant recruitment (hospital-based versus population-based study). Nonetheless, our results as a whole allow us to confirm and expand on the findings of previous reports. In a population of overweight/obese children and adolescents, even slightly elevated serum levels of TSH are associated with an increased risk for NAFLD.

There are several underlying mechanisms which may substantiate the relationship between thyroid dysfunction and hepatic steatosis. Thyroid hormones influence all major metabolic pathways, and thyroid dysfunction, especially hypothyroidism, has been associated with IR [24, 25], dyslipidemia [26, 27], and obesity [28, 29], all of which play an important role in the development of NAFLD. IR in the setting of hypothyroidism has been documented [25] and is associated with decreased responsiveness of glucose uptake in muscle and adipose tissue to insulin, as well as decreased glycogen synthesis in skeletal muscle in both animal and human studies [24, 25, 30, 31]. These effects were alleviated by thyroid replacement [24]. Recently, IR has been shown to also exist in SH and to be comparable to that of clinical hypothyroidism [32]. Another explanation for the association between thyroid function and NAFLD is that hypothyroidism is associated with abnormal lipid values [27]. Hypothyroidism primarily causes elevation in cholesterol and low-density lipoproteins but it also affects the synthesis, mobilization and degradation of all aspects of lipid metabolism [26]. The elevation of triglycerides in hypothyroid subjects is caused by a reduced removal rate of triglycerides from plasma due to a decrease in the activity of hepatic triglyceride lipase [26, 33]. The effects of SH on serum lipids values are less clear. While some studies have demonstrated that serum triglycerides, lipid subparticle size, and low-density lipoprotein cholesterol (LDL-C) oxidizability may be altered in patients with SH, others have not shown any effect on these lipid alterations [34]. In agreement with previous studies in children [35, 36], we found that elevated TSH was significantly associated with hypertriglyceridemia as well as IR, independent of total and central obesity. Finally, hypothyroidism has been reported to modulate mitochondrial nitric oxide synthesis and alter mitochondrial inner membrane composition and permeability which alters respiratory gene expression and mitochondrial oxygen uptake [37]. Such abnormalities would result in increased ADP concentration and generation of reactive oxygen species [38]. 

This study has a few potential limitations. First, we had no histopathological data. Nonetheless, liver biopsies in a large study collective such as our cohort are difficult to perform, also for ethical reasons, as no specific therapy follows histologic diagnosis of NAFLD apart from recommending lifestyle modification which is generally advised to all obese children. Conversely, ultrasonography is by far the most common way of diagnosing hepatic steatosis in clinical practice. Second, it is cross-sectional; thus, our data are associations and do not prove causality. As opposed to healthy controls, serum TSH concentrations have been consistently found to be higher in obese subjects [13, 39]. Unlike TSH, data regarding the circulating levels of free thyroid hormones are discrepant between different studies, which reported either increased or decreased serum concentrations of FT3 [13, 28, 40, 41], with normal or decreased FT4/FT3 ratios [28, 41]. Because in some studies TSH fell with weight loss [42], it has been suggested that higher TSH in obese subjects is simply a metabolic adaption to excess body fat. However, weight and/or fat loss does not predictably decrease TSH and T3 [43–45]. Others have suggested that obesity-related SH, characterized by an increased serum TSH concentration with normal concentrations of the thyroid hormones, may be associated with dyslipidemia, IR, subclinical inflammation, and increased risk for coronary heart disease [46, 47]. In the study by Aeberli et al. who prospectively examined the associations between changes in thyroid function, IR, and other metabolic risk factors in obese children undergoing weight and fat loss in a well-controlled 8-week inpatient program [35], changes in TSH did not correlate with losses of weight, fat, or lean tissue (as assessed by dual energy X-ray absorption), but they significantly correlated with fasting insulin and HOMA-IR, independently of body weight and body composition. Yet, in that study [35], at baseline variation of TSH, but not variations in body weight or body fat, was a significant predictor of triglycerides, total cholesterol, and LDL-C. Baseline TSH was also an independent predictor of fasting insulin and HOMA-IR. In accordance with Aeberli et al. [35], we found that elevated TSH is a significant predictor of lipid and glucose dysmetabolism as well as of hepatic steatosis, independently of the degree of total and central obesity. Nonetheless, the possibility that thyroid hormones and NAFLD share common genetic or environmental influences accounting for the observed association cannot be discounted [48]. It is also possible that hepatic steatosis affects thyroid function rather than the other way around [48]. A longitudinal study with a cohort of children with and without hepatic steatosis at baseline would help clarify this.

## Figures and Tables

**Table 1:** Baseline characteristics of 402 overweight/obese children according to serum TSH.

	Serum TSH		*P* value
	≤4 mIU/L (*n* = 314)	>4 mIU/L (*n* = 88)	
TSH, mIU/L	2.42 (2.33–2.52)	4.74 (4.52–4.95)	<0.0001
Age, years	10.7 (10.3–11.1)	10.9 (10.0–11.2)	0.46
Male gender, *n* (%)	175 (55.7)	55 (62.5)	0.35
Prepubertal, *n* (%)	159 (50.6)	58 (65.9)	0.56
Weight, Kg	61 (59–63)	60 (55–65)	0.70
Height, cm	149 (147–152)	149 (144–153)	0.80
BMI, Kg/m^2^	26.3 (25.7–26.8)	26.3 (25.2–27.3)	0.75
BMI-SDS	2.0 (1.94–2.2)	2.0 (1.9–2.2)	0.80
Waist circumference, cm	87 (84–90)	89 (87–90)	0.26
Systolic blood pressure, mmHg	109 (107–110)	110 (107–112)	0.88
Diastolic blood pressure, mmHg	69 (68–70)	70 (67–72)	0.82
Triglycerides, mg/dL	99 (90–108)	119 (103–135)	<0.01
Total cholesterol, mg/dL	160 (156–165)	176 (166–186)	<0.01
HDL-C, mg/dL	47 (46–49)	45 (43–48)	0.30
Fasting glucose, mmol/L	4.71 (4.64–4.78)	4.67 (4.58–4.75)	0.48
Insulin, mU/L	16.0 (15.0–18.0)	19.3 (16.5–22.0)	<0.05
HOMA-IR values	3.62 (3.22–4.0)	4.0 (3.4–4.6)	<0.05
ALT, IU/L	31 (28–35)	52 (36–68)	<0.01
GGT, IU/L	17 (16–19)	22 (18–26)	<0.01
FT3, pg/mL	4.26 (4.18–4.35)	4.35 (4.26–4.42)	0.14
FT4, ng/dL	1.18 (1.15–1.22)	1.18 (1.12–1.22)	0.76

Data are expressed as *n* (%), mean or geometric mean (95% confidence intervals).

TSH: thyroid-stimulating hormone; BMI: body mass index; BMI-SDS: BMI-SD score; HDL-C: high-density lipoprotein cholesterol; HOMA-IR: homeostasis model assessment of insulin resistance; ALT: alanine aminotransferase; GGT: gamma-glutamyl transferase; FT3: free triiodothyronine; FT4: free thyroxine.

**Table 2:** Prevalence of hepatic steatosis, hepatic steatosis with elevated ALT, metabolic syndrome, and its components according to serum TSH among 402 overweight/obese children.

	Serum TSH		*P* value
	≤4 mIU/L (*n* = 314)	>4 mIU/L (*n* = 88)	
Hepatic steatosis	101 (32.2)	43 (48.9)	<0.01
Hepatic steatosis with elevated ALT	73 (23.2)	31 (35.2)	<0.01
Central obesity	148 (47.1)	41 (46.6)	0.58
Elevated blood pressure	23 (7.3)	6 (6.8)	0.78
Hypertriglyceridemia	69 (22.0)	35 (39.8)	<0.01
Low HDL-C	57 (18.2)	17 (19.3)	0.20
Elevated total cholesterol	94 (29.9)	39 (44.3)	<0.01
Glucose ≥5.6 mmol/L	8 (2.5)	2 (2.3)	0.35
Insulin resistance	89 (28.3)	39 (44.3)	<0.01
Metabolic syndrome	32 (10.2)	17 (19.3)	<0.05

Data are presented as *n* (%).

TSH: thyroid-stimulating hormone; HDL-C: high-density lipoprotein cholesterol; ALT: alanine aminotransferase.

**Table 3:** Adjusted odds ratios (and 95% CIs) of hepatic steatosis, hepatic steatosis with elevated ALT, metabolic syndrome, and its components among 402 overweight/obese children.

	Serum TSH		*P* value
	≤4 mIU/L (*n* = 314)	>4 mIU/L (*n* = 88)	
Hepatic steatosis			
Adjusted OR (95% CI)^+,∗^	1.00 (referent)	2.10 (1.22–3.60)	<0.01
Hepatic steatosis with elevated ALT			
Adjusted OR (95% CI)^+,∗^	1.00 (referent)	2.42 (1.29–4.51)	<0.01
Central obesity			
Adjusted OR (95% CI)^+^	1.00 (referent)	1.32 (0.57–3.01)	0.52
Elevated blood pressure			
Adjusted OR (95% CI)^+,∗^	1.00 (referent)	1.31 (0.41–4.17)	0.58
Hypertriglyceridemia			
Adjusted OR (95% CI)^+,∗^	1.00 (referent)	2.58 (1.39–4.78)	<0.01
Low HDL-C			
Adjusted OR (95% CI)^+,∗^	1.00 (referent)	1.97 (0.98–3.98)	0.063
Elevated total cholesterol			
Adjusted OR (95% CI)^+,∗^	1.00 (referent)	2.23 (1.30–3.84)	<0.01
Glucose ≥5.6 mmol/L			
Adjusted OR (95% CI)^+,∗^	1.00 (referent)	0	
Insulin resistance			
Adjusted OR (95% CI)^+,∗^	1.00 (referent)	2.62 (1.45–4.74)	<0.01
Metabolic syndrome			
Adjusted OR (95% CI)^+,∗^	1.00 (referent)	2.55 (1.13–5.76)	<0.05

^+^Age, gender, pubertal status, and BMI-SDS as well as FT3 and FT4 were included in the model; *similar results were obtained when adjusting for WC (instead of BMI-SDS).

TSH: thyroid-stimulating hormone; OR: odds ratio; CI: confidence interval; ALT: alanine aminotransferase; HDL-C: high-density lipoprotein cholesterol; BMI-SDS: body mass index-SD score; FT3: free triiodothyronine; FT4: free thyroxine; WC: waist circumference.
